# Isolation, purification, and full NMR assignments of cyclopamine from *Veratrum californicum*

**DOI:** 10.1186/1752-153X-2-12

**Published:** 2008-06-24

**Authors:** John E Oatis, Pam Brunsfeld, James W Rushing, Peter D Moeller, Daniel W Bearden, Thomas N Gallien, George Cooper

**Affiliations:** 1 Department of Pharmacology & Experimental Therapeutics, Medical University of South Carolina, Charleston, SC 29425 USA; 2 University of Idaho Stillinger Herbarium, Moscow, ID 83844 USA; 3 Clemson University Coastal Research & Education Center, Charleston, SC 29414 USA; 4 Toxin/Natural Products Program, Center for Coastal Environmental Health & Biomolecular Research, National Oceanic & Atmospheric Administration – National Ocean Service, Charleston, SC 29412 USA; 5 National Institute of Standards and Technology, Hollings Marine Laboratory, Charleston, SC 29412 USA; 6 Gazes Cardiac Research Institute, Cardiology Division, Medical University of South Carolina, Charleston, SC 29403 USA

## Abstract

**Background:**

The Hedgehog signaling pathway is essential for embryogenesis and for tissue homeostasis in the adult. However, it may induce malignancies in a number of tissues when constitutively activated, and it may also have a role in other forms of normal and maladaptive growth. Cyclopamine, a naturally occurring steroidal alkaloid, specifically inhibits the Hedgehog pathway by binding directly to Smoothened, an important Hedgehog response element. To use cyclopamine as a tool to explore and/or inhibit the Hedgehog pathway *in vivo*, a substantial quantity is required, and as a practical matter cyclopamine has been effectively unavailable for usage in animals larger than mice.

**Results:**

In this paper, we report a rapid and efficient isolation and purification of large quantities of cyclopamine from the roots and rhizomes of *Veratrum californicum *Dur. (the Corn Lily or Western false hellebore). We also provide unambiguous assignments of the carbon and proton resonances by using the multinuclear spectra and the spin coupling networks.

**Conclusion:**

This method could meet a very real need within diverse scientific communities by allowing cyclopamine to become more readily available.

## Background

Cyclopamine is a naturally occurring steroidal alkaloid teratogen that interferes with the Hedgehog (Hh) signaling pathway by binding to Smoothened, a heptahelical transmembrane receptor that is a critical Hh response element [1,2]. Cyclopamine is thus a very useful reagent for probing and modifying the activity of the important and widely studied Hh pathway. As one important example, cyclopamine has been shown to be an extremely effective inhibitor of the growth and survival of specific tumors *in vitro *and *in vivo *[1,2], such that cyclopamine, cyclopamine analogs, and related alkaloids [3,4] have the potential to become important cancer chemotherapeutic agents. However, because it is very expensive commercially (~$10/mg), the large quantities required for research in animals, and especially in large mammals as required in our own cardiac research, would at present be prohibitively expensive.

A totally synthetic approach to obtaining cyclopamine can be envisioned. First, the synthesis of 17-acetyl-5α-etiojerva-12,14,16-trien-3β-ol from simple chemicals has been reported by Johnson et al [5], and Masamune et al reported [6] its conversion into the cyclopamine relative jervine, where the carbon-11 methylene in cyclopamine is oxidized to ketone in jervine. Second, the Wolff-Kishner reduction of jervine has been reported to yield cyclopamine [7]. However, because this would in aggregate be a nontrivial complex multistep synthesis of very low net yield, and because the concentration of cyclopamine in *Veratrum californicum *is so high, *vide infra*, we opted to isolate the compound from this natural source.

The isolation and purification procedures specifically designed for *Veratrum *alkaloids that have been reported previously [8-11] were also lengthy and of relatively low yield. Thus, in order to obtain the large amounts of compound necessary for our animal research we required a significantly more efficient method to extract and purify cyclopamine from the roots and rhizomes of *Veratrum californicum*. In this paper we report the rapid isolation and purification of cyclopamine with excellent yields. We also provide unambiguous assignments of the carbon and proton resonances by using the multinuclear spectra and the spin coupling networks.

## Results and discussion

### Overview of the methodology

In previous isolations [8-11], cyclopamine was extracted by allowing room-temperature benzene to percolate through dried, ground roots and rhizomes of *Veratrum californicum *which had been wetted with 5% ammonia. After storing the eluent at 5°C for several months, a mixture of alkaloids crystallized. Purification was then effected by multiple recrystallization steps to give a yield of about 320 mg per kilogram of dried roots.

Due to our need for significant quantities of cyclopamine, we required a practical large-scale isolation scheme. We felt that Soxhlet extraction utilizing refluxing benzene would represent a more efficient procedure than that reported at room temperature. Liquid chromatography-mass spectrometry (LC-MS) analysis demonstrated the efficacy of this method. This new method showed that very little cyclopamine remained in the plant material after the tenth Soxhlet extraction cycle.

This extraction procedure also allowed for quantitative determination of cyclopamine from the natural source. Quantitative LC-MS showed that our collection of roots and rhizomes of *Veratrum californicum *contained 2.34 g of cyclopamine per kg of dried plant material. Large-scale extractions were effected using two Soxhlet extractors fitted with a heating mantle, with benzene as the solvent. The largest cellulose extraction thimbles available to us were 90 × 200 mm and could hold only 0.70 kg of dried plant material. For this reason we adopted homemade cloth extraction thimbles, sewn from 100% cotton, to increase extraction throughput. The large volume of benzene (7.5 L for each of two Soxhlets) needed for these extractions precluded the use of normal evaporation techniques to obtain the crude product.

The next step in the process was to isolate the cyclopamine from the large volume of benzene. This was easily effected by passing the entire benzene solution through a large glass column charged with silica gel. Analytical silica gel thin layer chromatography (TLC) analysis demonstrated that benzene was not a sufficiently strong elution solvent to move the alkaloids from the origin. This effectively allowed us to trap all of the cyclopamine from the benzene solution while recapturing the benzene for subsequent Soxhlet extractions. To this end, the benzene from both Soxhlets was added to the top of a silica gel column fitted with a vacuum apparatus to speed the separation. Vacuum filtration allowed for the entire volume of benzene to pass through the column in approximately 45 min. This process resulted in concentrating the plant alkaloids (including the cyclopamine) at the top of the column. A remarkable advantage of this method was the useful result that the eluted benzene contained so little material that it could be reused repeatedly before a redistillation step for purification was required. To expedite the flash chromatography, the column was connected to a vacuum source to pull the solvent through the column. Elution of the alkaloids was effected using a step gradient of CH_2_Cl_2_-isopropanol (97:3), CH_2_Cl_2_-isopropanol (93:7), and then CH_2_Cl_2_-isopropanol (75:25). Fractions containing semi-pure cyclopamine were concentrated with reduced pressure. Re-chromatography of these semi-purified extracts on silica gel eluting with ethyl acetate-isopropanol-ammonia (85:14:1) yielded cyclopamine pure enough to solidify by trituration with cold acetone. Although significant amounts of product remained in the acetone wash, most of the impurities from the precipitate were removed, such that only two subsequent recrystallizations from EtOH-H_2_O (10:1) were required to give cyclopamine that contained no detectable impurities as measured by LC-MS analysis. The filtrates from multiple acetone washes and recrystallizations were pooled and then repurified by flash chromatography and recrystallization to obtain additional product.

### Structural analysis

**Figure 1:**
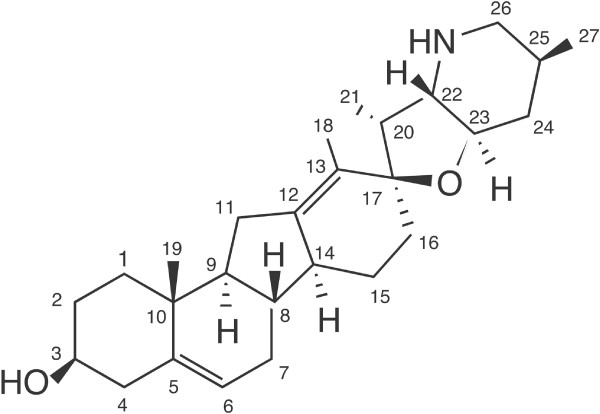
Cyclopamine.

The static 3-D structure of cyclopamine [see Additional file 1] is put into rotation [see Additional file 2] to elucidate its complex stereochemistry. Unambiguous assignments of the carbon and proton resonances were possible using the multinuclear spectra and the spin coupling networks. The resonance assignments are shown in Table 1 and the accompanying Figure 1. Chemical shifts are based on the use of the solvent deuterium resonance as internal standard with CD_2_Cl_2 _ at 5.320 ppm, resulting in the center of the ^13^C CD_2_Cl_2 _multiplet being at 53.438 ppm. The stereochemical assignments of methylene hydrogens shown in Table 2 [see Additional files 3, 4, 5] were made based on gradient NOESY cross-peaks and were consistent with distances determined from a computational, energy-minimized model of the molecule utilizing the MMX force-field (PCModel, version 9.2; Serena Software, Bloomington, IN). Over 30 NOE cross-peaks were identified and determined to be consistent with interatomic distances of less than 3 angstroms.

**Table 1:** 700 MHz Proton and Carbon-13 Chemical Shift Assignments ^a ^for Cyclopamine in CD_2_Cl_2 _at 25°C

Atom	Proton	Carbon
1 ax	1.23	38.2
1 eq	1.76	
2 ax	1.55	31.5
2 eq	1.83	
3	3.51	71.7
4 ax	2.23	41.9
4 eq	2.38	
5	-	141.9
6	5.42	121.6
7 ax	1.78	31.1
7 eq	2.27	
8	1.31	41.9
9	1.45	52.1
10	-	36.5
11 ax	2.18	28.8
11 eq	2.27	
12	-	142.2
13	-	127.1
14	1.72	49.1
15 ax	1.25	24.7
15 eq	1.81	
16 ax	1.51	31.9
16 eq	1.89	
17	-	85.0
Me-18	1.65	12.8
Me-19	1.00	18.4
20	2.43	49.9
Me-21	0.93	10.4
22	2.62	66.5
23	3.19	75.5
24 ax	1.14	39.1
4 eq	2.12	
25	1.59	31.7
26 ax	2.29	54.8
26 eq	3.04	
Me-27	0.95	18.7

^a^δ in ppm

**Table 2:** 700 MHz Proton-Proton Nuclear Overhauser Interaction Peaks ^a ^for Cyclopamine in CD_2_Cl_2 _at 25°C

No.^b^	Atom 1	Atom 2	Relationship
1	1 ax	3	ax-ax
2	1 ax	9	ax-ax
3	2 ax	4 ax	ax-ax
4	2 ax	19	ax-ax
5	4 eq	6	eq-eq
6	8	19	ax-ax
7	8	7 e	ax-eq
8	8	15 ax	ax-ax
9	9	7 ax	ax-eq
10	9	14	ax-ax
11	16 eq	23	eq-ax
12	16 ax	14	ax-ax
13	18	20	eq-eq
14	18	22	eq-ax
15	19	1 ax	ax-ax
16	19	4 ax	ax-ax
17	19	11 ax	ax-ax
18	20	22	eq-ax
19	21	16 eq	ax-eq
20	21	16 ax	ax-ax
21	21	23	ax-ax
22	24 eq	23	eq-ax
23	24 ax	18 (vw^c^)	ax-eq
24	24 ax	26 ax	ax-ax
25	24 ax	22	ax-ax
26	24 ax	23 (vw^c^)	ax-eq
27	26 ax	22	ax-ax
28	25	23	ax-ax
29	25	24 eq	ax-eq
30	25	26 eq	ax-eq
31	27	24 eq	eq-eq
32	27	24 ax	eq-ax
33	27	26 eq	eq-eq
34	27	26 ax	eq-ax
35	20	16 ax (vw^c^)	eq-ax
36	8	11 ax	ax-ax

^a^Only the NOEs that were used to obtain hydrogen and stereochemical assignments are included.

^b^Numbering scheme corresponds to NOESY peaks identified in Additional files 3, 4, 5.

^c^very weak

The analysis not only shows general agreement with previous partial assignments [12], but our assignments are complete. In addition, the assignments of C-12 and C-13 are corrected, and C-2 and C-16 are disambiguated.

## Conclusion

We have optimized the isolation, the purification, and the yield of cyclopamine from the roots and rhizomes of *Veratrum californicum*. This procedure is readily scalable to produce the quantities required for *in vivo *and *in vitro *research, for potential clinical trials, or for conversion to derivatives that are more potent inhibitors of Smoothened and that may have better oral bioavailability.

## Experimental

### General comments

All extractions were carried out in a closed system consisting of a Soxhlet extractor equipped with a chilled condensor (-4°C). Benzene was determined to be an efficient extraction solvent and was easily recaptured and reused. The extraction apparatus was fitted into a large walk-in hood to ensure safe ventilation of any benzene vapor that might escape from the extraction unit. For the first flash chromatography separation, Fisher 70–230 mesh silica gel with a 60 Å pore size was used; the second chromatographic step employed J.T. Baker 40 μm silica gel flash chromatography packing with a 60 Å pore size. The progress of each separation was followed by TLC on 5 × 10 cm, 250 μm TLC plates (EMD Chemicals, Darmstadt, Germany) using EtOAc-isopropanol-NH_4_OH, 80:14:1 (rf of cyclopamine 0.24). After development, the spots were visualized by staining with phosphomolybdic acid.

### HPLC-MS

Cyclopamine samples were analyzed via LC-electrospray ionization (ESI)-tandem mass spectrometry (MS/MS) on a quadrupole ion trap mass spectrometer (LCQ Classic, Thermo-Finnigan, San Jose, CA) coupled to a Hewlett-Packard 1100 HPLC system. A 1.2 × 100 mm XTerra MS C-18 3.5 μm C-18 reversed-phase LC column (Waters Corporation, Milford, MA) was utilized. Cyclopamine was eluted employing a 30 min gradient starting at 10% acetonitrile, 0.2% aqueous formic acid to 70% acetonitrile, 0.2% aqueous formic acid with a flow rate of 100 μL/min and a 1:10 post-column split. A blank was analyzed between samples to limit carryover. Some mass spectra were also obtained either on a Waters Micromass ZQ Mass detector equipped with a Waters 600 HPLC pump and photodiode array detector or on a Thermo-Finnegan TSQ Quantum equipped with an Agilent 1100 series HPLC system.

### Nuclear magnetic resonance spectroscopy

Initial NMR spectra of cyclopamine were acquired on a 400 MHz spectrometer (Varian ^UNITY^*INOVA*, Palo Alto, CA) equipped with a 5 mm indirect detect z-gradient probe in 700 μL deuteromethylene chloride (CD_2_Cl_2_). Partial assignments of the proton spectra were achieved by employing a DQCOSY experiment acquired in the phase-sensitive mode. The chemical shifts of unresolved multiplets were based on the chemical shifts of the cross-peaks. Partial assignments of the carbon resonances were made using gradient versions of multiplicity-edited HSQC, and gradient HMBC experiments were used for long-range correlations. Some proton assignments were made and stereochemical relationships were established by employing NOESY.

High-resolution NMR spectra were collected on a 700 MHz spectrometer (Avance II, Bruker Biospin, Inc., Billerica, MA) equipped with a triple-resonance, z-gradient cryogenic probe (5 mm TCI Cryoprobe, Bruker Biospin, Inc.). A 6.8 mg sample was dissolved in 600 μL CD_2_Cl_2 _and analyzed at 298°K. In addition to standard proton and carbon spectra, a number of two-dimensional experiments were conducted in order to fully assign the resonances. Homonuclear assignments were aided by gradient phase-sensitive, DQCOSY spectra and gradient phase-sensitive NOESY spectra. Heteronuclear correlations were determined by using gradient multiplicity-edited, sensitivity-enhanced HSQC correlation spectroscopy, and long-range correlations were determined using gradient HMBC. Multiplicity (CH_n_, n = 0–3) was confirmed through one-dimensional DEPT pulse sequences. Spectra were processed using the instrument software (TopSpin, Bruker Biospin, Inc.), and analysis was conducted using iNMR (Nucleomatica, Molfetta, Italy; ), a computer program designed to aid in resonance assignments.

### Cyclopamine isolation

*Veratrum californicum *is an abundant, robust perennial herb 1.5 to 2.0 meters in height that is commonly found at elevations of 1500 to 4000 meters along open watercourses and in moist meadows of the northern Rocky and Pacific coastal mountains. Roots and rhizomes from this plant, wherein steroidal alkaloids are concentrated, were collected in Idaho's Clearwater National Forest during September 2005. They were thoroughly air dried, milled to a coarse powder (Triarco Industries, Green Pond, SC), and stored in sealed containers with desiccant at 0°C. For each 130 × 300 mm Soxhlet extractor (Southeastern Lab Apparatus, North Augusta, SC), 1.45 kg of powdered plant that had been wet with 800 mL of 7.5% aqueous NH_4_OH (3.4 mole) was placed in a 110 × 280 mm 100% cotton cloth bag. Each bag was extracted for 14 hrs with 7.5 L of refluxing benzene. After cooling to room temperature, the combined benzene solutions were chromatographed on 1.5 kg of silica gel in a 16 cm column and the solvent pulled through with vacuum. Evaporation of 100 mL aliquots from 10 runs indicated that an average of 140 g of alkaloids was put on the column. The column was freed of benzene, washed with 4 L of CH_2_Cl_2_-isopropanol (97:3), and then washed again with 10 L of CH_2_Cl_2_-isopropanol (93:7). Fractions highly enriched in cyclopamine were then eluted with CH_2_Cl_2_-isopropanol (75:25) in the next 10 L. After the solvents were removed at reduced pressure, an average of 50 g of semi-pure cyclopamine per extraction cycle was obtained. For the second chromatography step, 100 g of semi-pure cyclopamine dissolved in 500 mL of CH_2_Cl_2 _was absorbed on 300 g of silica gel, and the solvent was removed at reduced pressure. The resulting dry power was placed on top of 2.5 kg of dry silica gel in a 16 cm column, and the column was wet with 3 L of EtOAc. After washing the column with an additional 3 L of EtOAc, cyclopamine was eluted with EtOAc-isopropanol-NH_4_OH (85:14:1), with 1 L fractions being collected. Concentration of the fractions containing cyclopamine gave an average 23.3 g of a yellow-glass. After triturating with cold acetone (1.5 mL/g) and cooling at 0°C for at least 18 hrs, filtration yielded an average of 10.1 g of a cream solid. Two recrystallizations of 29.0 g from EtOH-H_2_O (10:1), 8 ml/g, gave 21.7 g of cyclopamine with a purity >99%. From the primary isolation and purification, 1.3 g per kilogram of dried root, or 55% of the available cyclopamine was recovered. Repurification of all of the acetone wash filtrates as well as the filtrates from the ethanol recrystallizations would substantially increase the percent recovery.

The 400 MHz NMR spectrum and the ESI mass spectrum of this isolated product were identical to those of reference cyclopamine (a kind gift from Dale R. Gardner, Poisonous Plants Research Lab, Logan, UT). Benzene recovered from the column could be used repeatedly for subsequent extractions before purification by redistillation was required. The methylene chloride was also reused after drying over calcium sulfate and fractional distillation.

## Authors' contributions

PB, JR, TG, and GC were responsible for gathering and processing the roots and rhizomes of *Veratrum californicum*. JO, PM, DB, TG, and GC were responsible for the extraction and structural analysis of cyclopamine. JO, DB, and GC were responsible for the preparation of the manuscript. All authors read and approved the final manuscript.

## Supplementary Material

Additional file 1 Cyclopamine 3-D. Cyclopamine depicted as a ball-and-stick image with perspective. The energy-minimized structure of cyclopamine (energy-minimized using the MMX force-field [PCModel, version 9.2; Serena Software, Bloomington, IN]) is shown after visual rendering with POV-Ray 3.6 for Mac OS (Build 197, Copyright^© ^1991–2003 Persistence of Vision Team™, Copyright^© ^2003–2004 Persistence of Vision Raytracer Pty. Ltd.; ). The input file for POV-Ray was generated by PCModel commands. The energy-minimized structure was confirmed with the global conformation searching utility in PCModel (GMMX), using random variation of the structure to generate candidate structures that were then minimized and compared to previously determined low-energy structures. Click here for file

Additional file 2 Cyclopamine Fixed x-y. Rotating cyclopamine structure shows the stereochemistry in detail. This is the same molecule as that shown in Figure S1. The X-Y rotation was generated using PCModel graphical output commands. Click here for file

Additional file 3 NOESY cross-peaks. The NOESY cross-peaks used in structural confirmation and assignments. The numbering of the cross-peaks is that shown in Table 2. A) Upfield region. B) Mid-field region. C) Low-field region. D) Full spectral window. Click here for file

Additional file 4 DQCOSY + NOESY. Overlay of DQCOSY on top of NOESY spectrum. The unique NOESY peaks are shown in brown and orange, and the COSY peaks are shown in magenta and cyan. A) Upfield region. B) Full spectral window. Click here for file

Additional file 5 Heteronuclear spectroscopy. Heteronuclear spectroscopy detail. A) The HSQC spectrum shows weak long-range coupling which disambiguates highly overlapped proton resonances of H-2 and H-16. The long-range peaks show C-2 to H-1 and C-16 to H-15 connectivities. B) The HMBC spectrum shows a long-range cross-peak between C-13 and H-20. Click here for file
