# Supplementary figures and images for: Isolation, purification, and full NMR assignments of cyclopamine from Veratrum californicum
Source: Chem Cent J. 2008 Jun 24;2:12. doi: 10.1186/1752-153X-2-12 (PMC2442831; doi:10.1186/1752-153X-2-12)

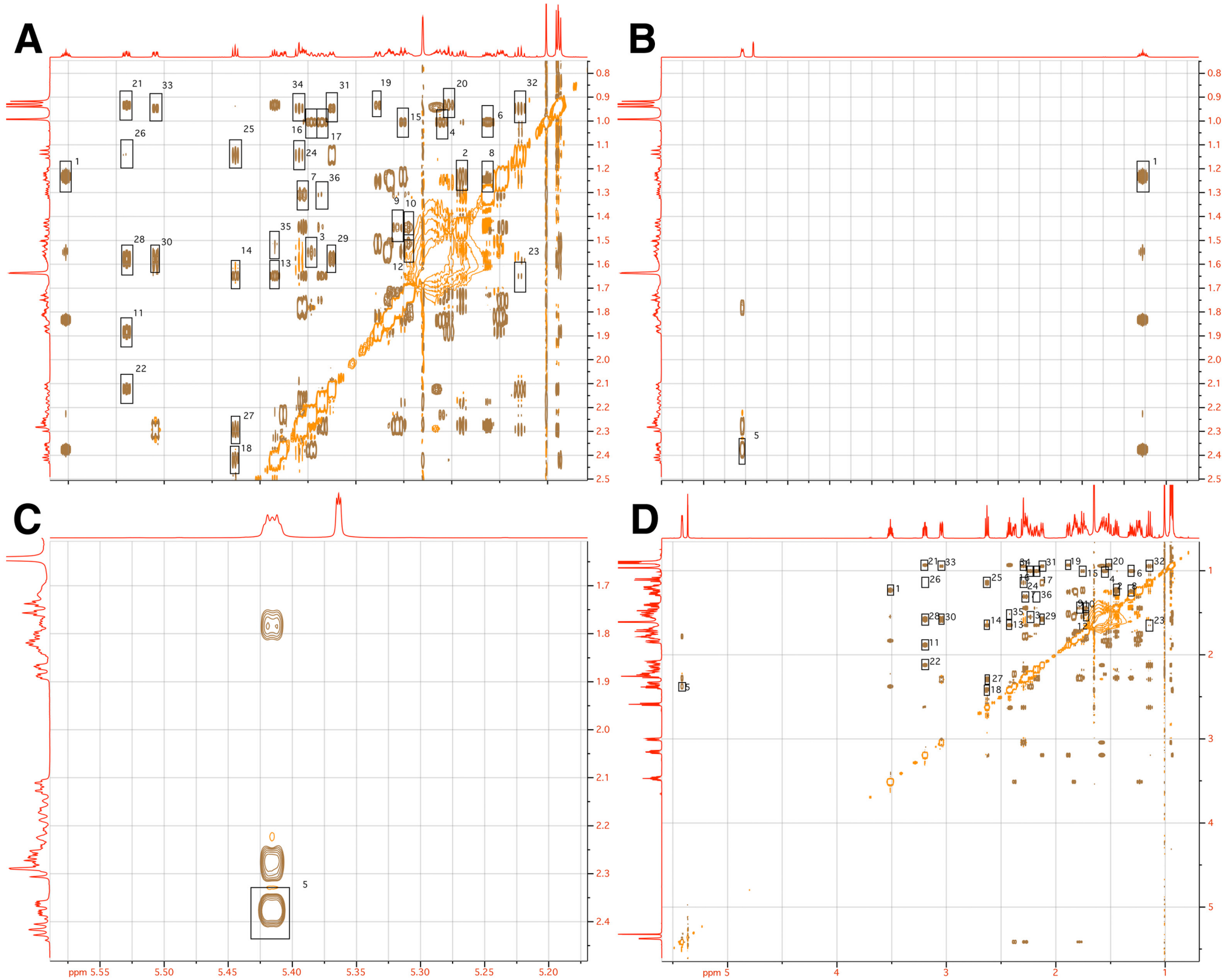

Supplement: Additional file 3 — NOESY cross-peaks. The NOESY cross-peaks used in structural confirmation and assignments. The numbering of the cross-peaks is that shown in Table 2. A) Upfield region. B) Mid-field region. C) Low-field region. D) Full spectral window. [file 1752-153X-2-12-S3.pdf]

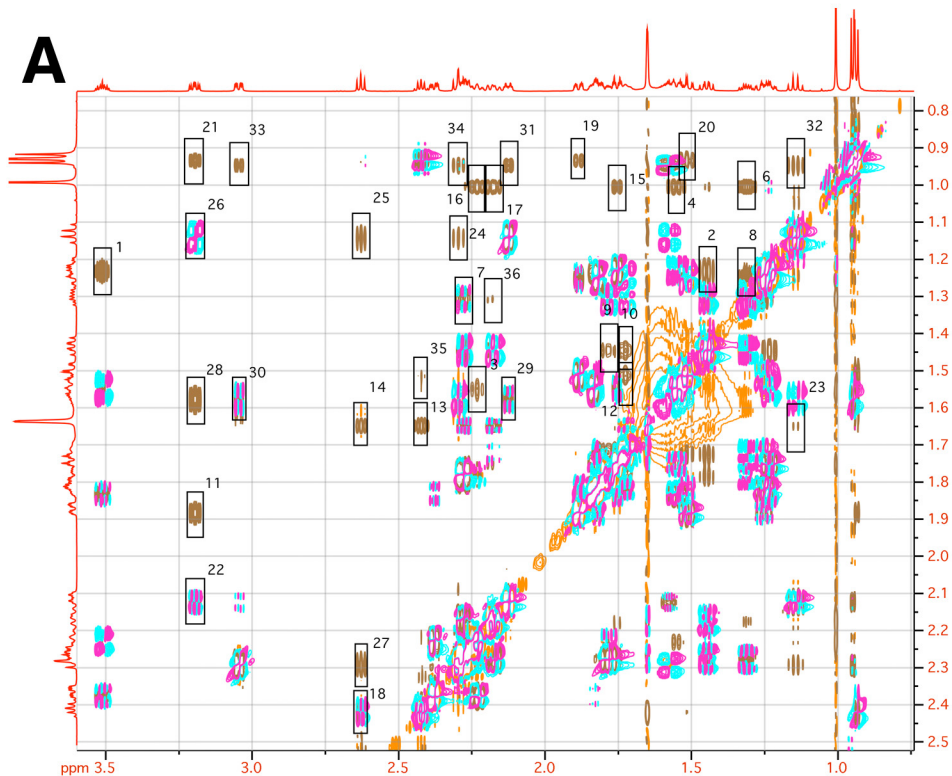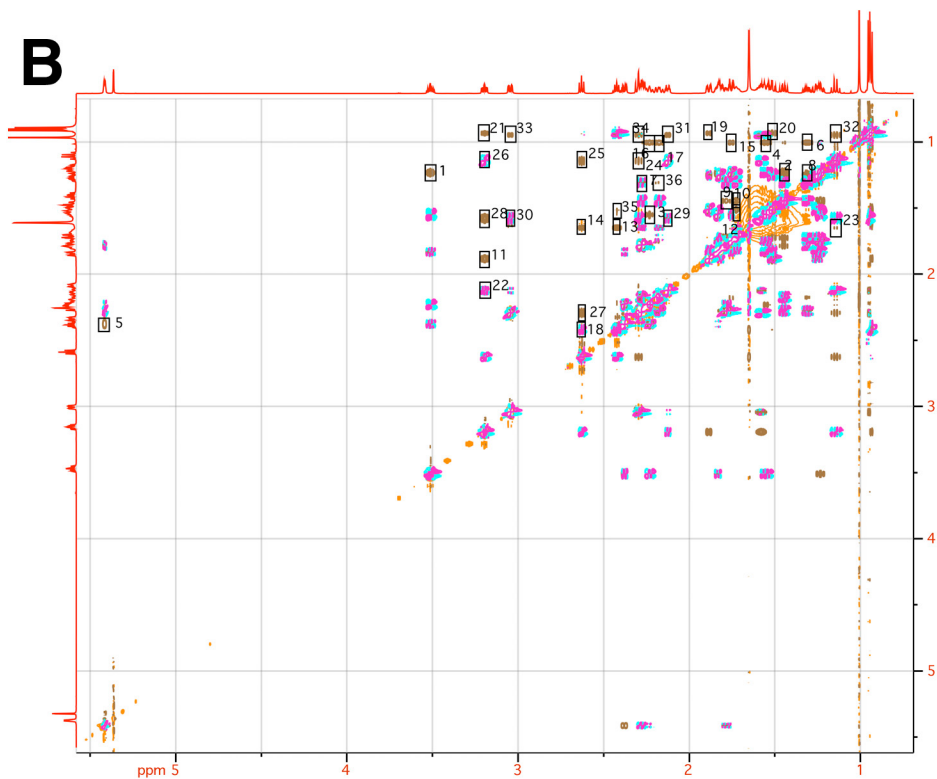

Supplement: Additional file 4 — DQCOSY + NOESY. Overlay of DQCOSY on top of NOESY spectrum. The unique NOESY peaks are shown in brown and orange, and the COSY peaks are shown in magenta and cyan. A) Upfield region. B) Full spectral window. [file 1752-153X-2-12-S4.pdf]

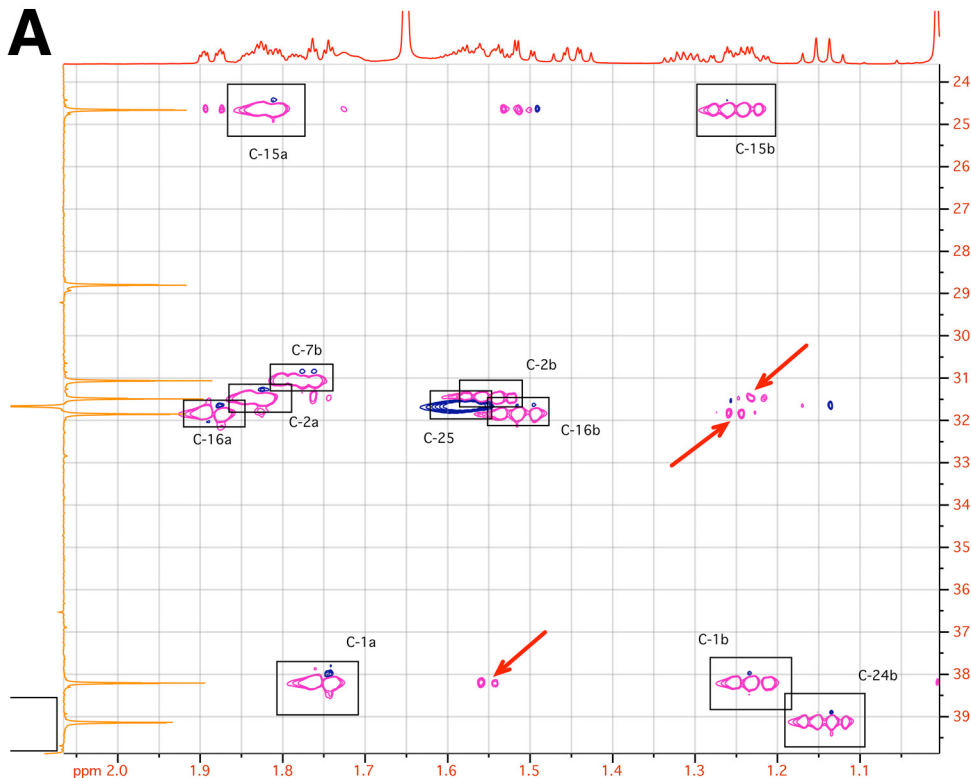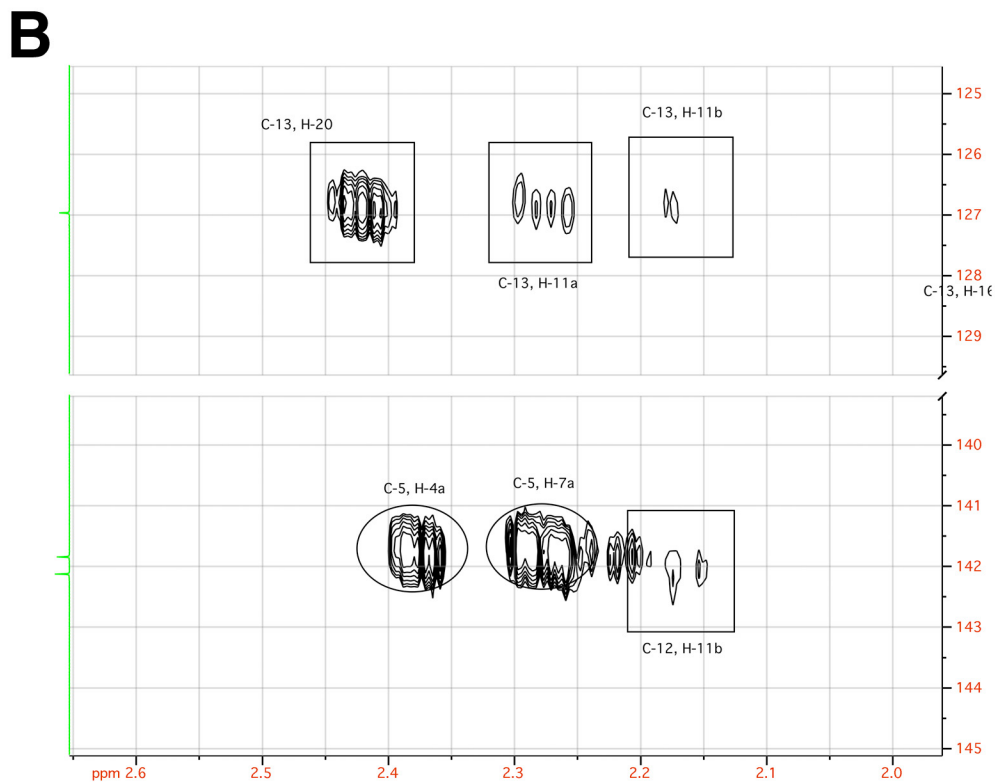

Supplement: Additional file 5 — Heteronuclear spectroscopy. Heteronuclear spectroscopy detail. A) The HSQC spectrum shows weak long-range coupling which disambiguates highly overlapped proton resonances of H-2 and H-16. The long-range peaks show C-2 to H-1 and C-16 to H-15 connectivities. B) The HMBC spectrum shows a long-range cross-peak between C-13 and H-20. [file 1752-153X-2-12-S5.pdf]
